# Effects of Polysaccharide Concentrations on the Formation and Physical Properties of Emulsion-Templated Oleogels

**DOI:** 10.3390/molecules27175391

**Published:** 2022-08-24

**Authors:** Zongbo Jiang, Xinpeng Bai

**Affiliations:** 1 School of Food Science and Engineering, Hainan University, No. 58 Renmin Avenue, Haikou 570228, China; 2 Engineering Research Center of Utilization of Tropical Polysaccharide Resources, No. 58 Renmin Avenue, Haikou 570228, China

**Keywords:** hydroxypropyl methyl cellulose, pectin, oleogels, physical properties, emulsion-templated method

## Abstract

An emulsion template method was an effective way to prepare oleogels. However, there were few reports on how hydroxypropyl methylcellulose-pectin (HPMC-PC) mixtures affected the physicochemical properties of the obtained oleogels. In this study, the oleogels were prepared by an emulsion template method. The influences of HPMC and PC concentrations on the formation and physical properties of the emulsions and oleogels were investigated, by analyzing particle size distribution, microstructure, rheological test, oil loss, and crystallinity. The results of particle sizes and microstructure showed that a high concentration of HPMC and PC exhibited a better emulsification performance. The rheological tests indicated that a high concentration of HPMC and PC contributed to an increase in the mechanical strength of emulsions and oleogels. Moreover, an increase in an HPMC and PC concentration was beneficial to reduce the oil loss of oleogels. However, the change of HPMC and PC concentrations had no significant effect on the X-ray diffraction pattern of oleogels. This study could provide a theoretical basis for the construction of polysaccharide-based oleogels.

## 1. Introduction

Obesity caused by a high intake of fat increased the risk of diabetes and cardiovascular disease [1]. Therefore, control of the amount and type of dietary fat was critical to prevent obesity. Medium-chain triglycerides (MCTs) were composed of medium chain fatty acids such as caprylic and capric acids. In contrast to long-chain triglycerides (LCTs), MCTs were easily hydrolyzed and absorbed and were not reconstituted into triglycerides in enterocytes for fat storage, thereby reducing the incidence of obesity [2]. However, the direct addition of MCT oil, liquid at ambient temperature, to foods is challenging. Thus, its conversion into an oleogel could enlarge its food applications.

An oleogel could be regarded as an organic liquid trapped in a three-dimensional network, which had the features of solid fat (rheological properties, viscoelasticity, consistency, etc.). Lots of small molecule surfactants, such as waxes [3], monoglycerides [4], lecithin [5], and phytosterols [6] acted as gelators to gel the liquid oils by a direct method. However, small molecule surfactants with high cost, large dosage, and large environmental pollution were unable to reach the requirements of the modern food industry for green and healthy food [7]. Polysaccharides have attracted a lot of attention because of their biocompatibility, biosafety and biodegradability, which were widely adopted in the food industry as emulsifiers, thickeners, and gelators [8]. However, most food polysaccharides are hydrophilic in nature and cannot directly form a gel network in liquid oils (hydrophobic solvent) except for modified chitin and ethyl cellulose [9]. Therefore, oleogel prepared by polysaccharides usually is achieved by an indirect method, such as the emulsion-template, foam-template, and aerogel-template method [10]. For example, gelatin and xanthan gum structured low-viscosity liquid oils into oleogels by an emulsion templating method. The results showed that increasing the concentration of gelatin and xanthan gum could improve the strength of the oleogels [11]. Sodium caseinate and hydroxypropyl methylcellulose (HPMC) were used to prepare oleogels by a foam template method. The results showed that the concentration of sodium caseinate and HPMC was the main factor determining the structure of the oleogels, and the obtained oleogels had ideal texture properties [12].

HPMC is an amphiphilic polysaccharide with both hydrophobic methoxy groups and hydrophilic hydroxypropyl groups. HPMC exhibits good surface activity and can be used to prepare oleogels through the network structure. The oleogels constructed by HPMC in combination with thickening agents exhibited better gel properties, compared with those using a single component HPMC [13]. Jiang et al. used HPMC and xanthan gum (XG) to prepare oleogels via a foam-templated method. The results showed that the addition of XG significantly improved the stability of the bubbles and the oil absorption capacity of the oleogels, and obtained oleogels exhibited high viscoelasticity and stability [14]. Espert et al. obtained the sunflower oil-bleased oleogels by an emulsion template method using methyl cellulose (MC) and HPMC as a unique oil gelling agent (without adding thickeners). The results showed that a higher cellulose concentration resulted in harder and more stable oleogels [15]. 

Pectin (PC) is an acidic heteropolysaccharide extracted from plant cell walls and is commonly used for thickening, gelling, and emulsification [16]. Most of the unesterified carboxyl groups in pectin molecules exist in the form of partially ionized salts, which can combine with hydroxyl groups to electrostatically adsorb in an oil/water interface layer, thereby reducing the interfacial tension and preventing droplet aggregation [17]. Moreover, it has been found that PC can enhance the stability of emulsions by providing steric repulsion and electrostatic interactions [18]. However, to the best of our knowledge, information on the construction of oleogels by PC with other polysaccharides (such as HPMC) is lacking. Therefore, the effect of HPMC-PC mixtures on the formation of oleogels needs to be further studied. 

In this work, the microstructure, rheological properties, oil retention, and X-ray diffraction of emulsions and oleogels were characterized to investigate the effects of polysaccharide concentrations on the formation and physical properties of oleogels. It could provide a theoretical basis for the practical application of oleogels in the food industry.

## 2. Results and Discussion

### 2.1. Microstructures and Particle Size of Emulsions

The small droplets often aggregated with each other to form large oil droplets during the storage process of the emulsion system, resulting in the instability of emulsions. The effect of HPMC or PC concentrations on emulsions was investigated by analyzing microstructure and particle size distributions in this work. For emulsions where the concentration of HPMC was varied, the concentration of PC was kept constant at 0.1 wt%. For emulsions where the concentration of PC was varied, the concentration of HPMC was kept constant at 0.8 wt%. When an HPMC concentration was lower than 0.4 wt%, the phase separation phenomenon occurred during the drying procedure; when the concentration was higher than 1.0 wt%, HPMC particles were difficult to fully hydrate in the aqueous solution. The HPMC concentration in this experiment was selected from 0.4 wt% to 1.0 wt%. 

**Figure 1:**
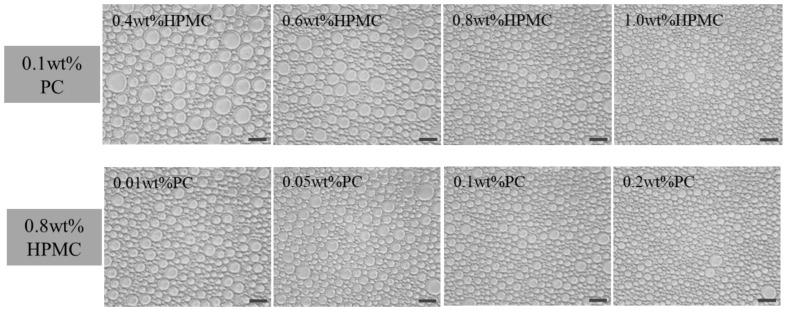
The microstructures of emulsions prepared by different HPMC and PC concentrations. For emulsions where the concentration of HPMC was varied, the concentration of PC was kept constant at 0.1 wt%. For emulsions where the concentration of PC was varied, the concentration of HPMC was kept constant at 0.8 wt%. The ruler is 20 μm. (HPMC–Hydroxypropyl methylcellulose, PC—Pectin).

**Figure 2:**
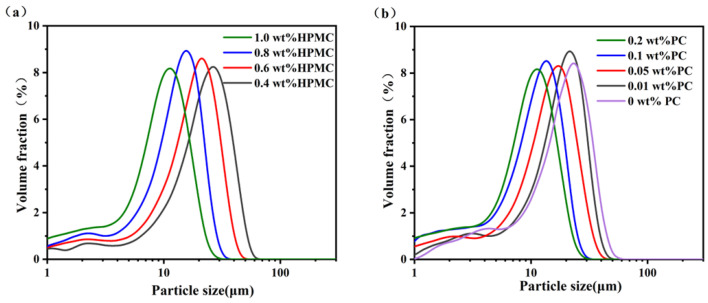

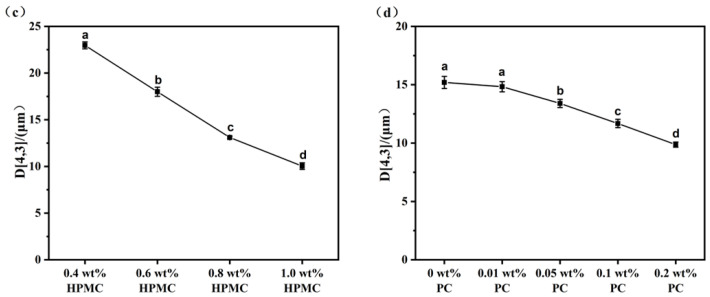
Droplet size distribution curves (**a**) and volume-average diameters (D[4,3]) (**c**) of different concentrations of HPMC emulsions, when the PC concentration was constant at 0.1 wt%. Droplet size distribution curves (**b**) and volume-average diameters (D[4,3]) (**d**) of different concentrations of PC emulsions, when the HPMC concentration was constant at 0.8wt%. Letters a–d indicate significant difference in different emulsions (*p* < 0.05). (HPMC—Hydroxypropyl methylcellulose, PC—Pectin).

As shown in Figure 1, there is no obvious coalescence in the emulsions, which may be related to the interfacial layer formed by HPMC-PC mixtures on the surface of oil droplets. Moreover, an increase in HPMC concentration reduced the average particle sizes of oil droplets from 22.9 μm to 10.1 μm, as shown in Figure 2c. It might be due to the fact that more HPMC adsorbed onto the oil-water interface facilitated to form small oil droplets [19]. Meng et al. also have similar findings where the average diameters of oil droplets gradually decreased with an increase in polysaccharide concentration [20]. Without changing the HPMC concentrations, the PC concentration was adjusted to 0–0.2 wt% to form emulsions. The particle size of the emulsion decreased from 15.2 μm to 9.8 μm with an increase in PC concentration (Figure 2b,d). This might be because the lower concentration of PC provided weaker electrostatic repulsion and steric hindrance resulting in larger oil droplet size, while the electrostatic interaction between a higher concentration of PC and HPMC formed multi-layer adsorption on the surface of oil droplets, which resisted the aggregation and coalescence of droplets [21]. The above results indicated that the increase in polysaccharide concentration could reduce the size of oil droplets. Patel et al. also suggested that high concentrations of polysaccharides had better emulsifying properties, which could form emulsions with better stability [11].

### 2.2. Characterization of Rheological Properties of Emulsions

**Figure 3:**
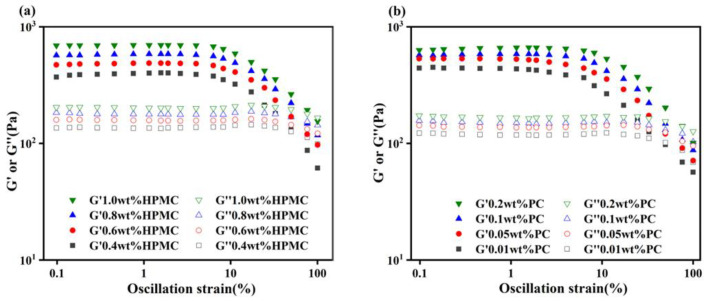

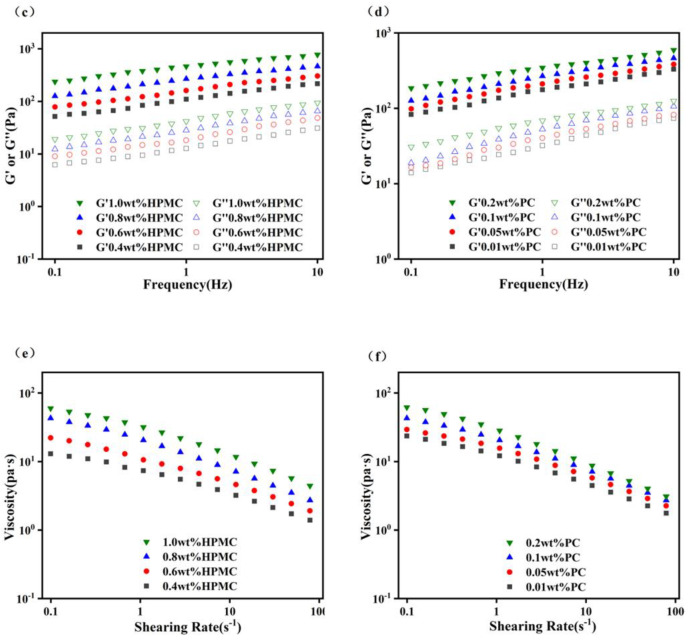
Amplitude sweep (**a**), frequency sweep (**c**), and flow measurement (**e**) curves for emulsions with different HPMC concentrations; a concentration of PC was kept constant at 0.1 wt%. Amplitude sweep (**b**), frequency sweep (**d**), and flow measurement (**f**) curves for emulsions with different PC concentrations; a concentration of HPMC was kept constant at 0.8 wt%. (HPMC—Hydroxypropyl methylcellulose, PC—Pectin).

The rheological properties of emulsions were further measured using a small-amplitude oscillatory shear (SAOS) rheometer. As shown in Figure 3a,b, the storage modulus (G′) values were always higher than the loss modulus (G″) values for all emulsions, reflecting the “solid-like” rheological behavior. Moreover, with a constant PC concentration (0.1 wt%), the increase in HPMC concentrations always increased the values of G′ and G″, indicating that a high concentration of emulsifier-facilitated emulsions behave as gels. As shown in Figure 3c,d, all emulsions exhibited a low dependence of G′ and G″ on the frequency. The emulsions with a higher HPMC concentration had thicker interfacial layers for stronger mechanical strength compared with those with a low concentration HPMC. This was due to the fact that emulsions with smaller particle sizes had a denser distribution of oil droplets, resulting in enhanced interaction between the droplets, which imparted stronger mechanical strength to the emulsions [22]. The study of Kim et al. also confirmed that the reduction in droplet size resulted in the increase in mechanical properties of the emulsions [23]. The increase in PC concentration was also accompanied by an increase in G′ and G″ values. As shown in Figure 3e,f, all samples exhibited the shear-thinning behavior. This was due to the directional alignment of oil droplets in the emulsion at high shear rates, reducing flow resistance [24]. It could be seen that the increase in HPMC and PC concentrations could both increase the apparent viscosity of the emulsions from the viscosity value of the emulsions at the initial shear rate (0.1 s^−1^).

### 2.3. Rheological Characterization of Oleogels

**Figure 4:**
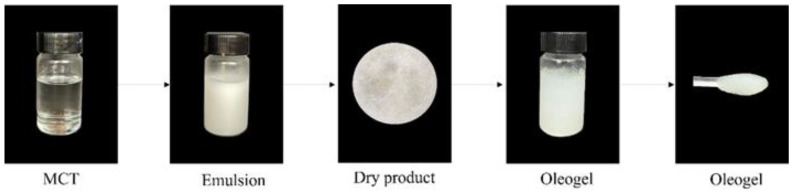
The process of preparation for oleogels.

The process of preparing oleogels by the emulsion template method was shown in Figure 4. No demulsification or oil leakage occurred during the dehydration process, proving that the emulsion was a good template for obtaining oleogels. It was worth noting that the spatial structure formed by HPMC-PC would be destroyed at a higher shear rate and longer shear time, resulting in a large amount of liquid oil leakage. Therefore, the oleogels were obtained by shearing the dried materials in this experiment.

**Figure 5:**
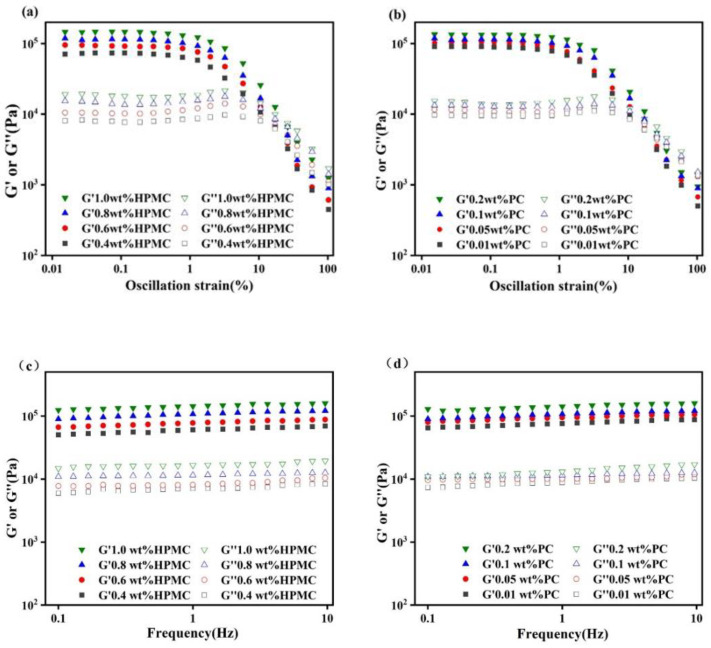

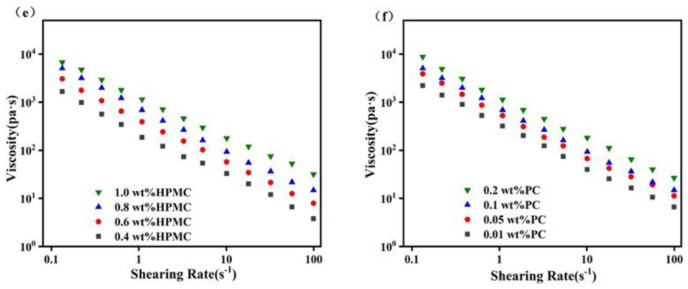
Amplitude sweeps (**a**), frequency sweeps (**c**) and flow measurement (**e**) for oleogels prepared by different HPMC concentrations (0.4, 0.6, 0.8 and 1.0 wt%) and a constant PC concentration (0.1 wt%). Amplitude sweeps (**b**), frequency sweeps (**d**) and flow measurement (**f**) for oleogels prepared by different PC concentrations (0.01, 0.05, 0.1 and 0.2 wt%) and a constant HPMC (0.8 wt%) concentration. (HPMC—Hydroxypropyl methylcellulose, PC—Pectin).

As shown in Figure 5a,b, in the linear viscoelastic region, the G′ value of the oleogels increased with an increase in polysaccharide concentration. This might be due to the fact that a higher HPMC concentration increased the network density and hardness of oleogels, resulting in oleogels with viscoelasticity [14], a higher pectin concentration increased the electrostatic repulsion between oil droplets, leading to an increase in the storage modulus [25]. A similar conclusion was drawn from this study [14] that the dense network structure formed in the oleogels obtained with a high concentration of polysaccharide possessed a great confinement ability for oil, resulting in a significant increase in G′ value. As shown in Figure 5c,d, the mechanical strength of all oleogel samples did not change significantly during the frequency change, indicating that the dependence of G′ on frequency was weak. As shown in Figure 5e,f, all the viscosities of oleogels decreased continuously as the shear rates increased (0.1–100 s^−1^), proving that the oleogels were pseudoplastic fluids [26]. Moreover, a higher HPMC and PC concentration resulted in the oleogels with higher apparent viscosity. Huang and Meng et al. also found that polymer concentration had an important impact on the viscoelasticity of oleogels, and they concluded that higher concentrations of polymers led to the oleogels with higher mechanical strength and better oil binding capacity [27,28]. Furthermore, the G′ values of the oleogels in this experiment were around 100,000 Pa, which were higher than those oleogels prepared with other gelling agent such as protein-polysaccharide mixtures, ethyl cellulose or wax [29,30,31].

**Figure 6:**
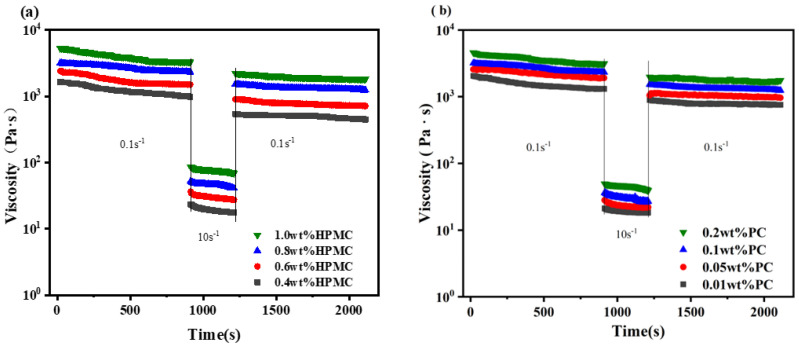
Time sweep (**a**) curves for oleogels prepared by different HPMC concentrations and a constant PC concentration (0.1 wt%). Time sweep (**b**) curves for oleogels prepared by different PC concentrations and a constant HPMC concentration (0.8 wt%). (HPMC—Hydroxypropyl methylcellulose, PC—Pectin).

In previous reports, the rheological properties of oleogels could vary greatly with the deformation of the structure [32,33]. Therefore, it is necessary to measure the structural recovery and thixotropy of the oleogel samples. As shown in Figure 6a,b, the viscosity of all oleogels decreased with time, indicating that the viscosity change of the samples was not only related to the shear rate, but also to the time of being sheared. The viscosity decreased immediately as the shear rate was changed from 0.1 s^−1^ to 10 s^−1^, which meant that the connections between the oleogel particles could be broken with sufficient force, resulting in a decrease in flow resistance [27]. Mert et al. found the same result by studying the oleogel obtained from xanthan gum and pectin [25]. Moreover, it was found that the structure recovery rate of all oleogel samples reached 70~90% through the comparison of the first and third time periods, indicating that the oleogels had strong structure recovery performance. From this, it could be seen that the structural recovery of HPMC-PC-based oleogels was better than those of wax-based oleogels because the structural recovery of wax-based oleogels was very limited when deformation occurred [34]. 

### 2.4. Oil Loss

**Figure 7:**
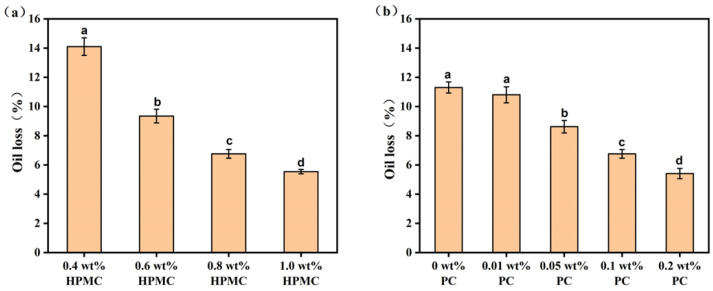
Oil loss of oleogels prepared by different HPMC concentrations and a constant PC concentration (0.1 wt%) (**a**). Oil loss of oleogels prepared with different PC concentrations and a constant HPMC concentration (0.8 wt%) (**b**). Letters a–d indicate significant difference in different oleogels (*p* < 0.05). (HPMC–Hydroxypropyl methylcellulose, PC—Pectin).

A lower oil loss rate (OLR) is a desirable property of oleogels, which means a high ability to retain oil in its structure and suitable physical stability. The OLR of different oleogels were shown in Figure 7. The OLR of all oleogels decreased with increasing polysaccharide concentration. The OLR as high as 14.1% occurred in the oleogel with 0.4 wt% HPMC, which was because the network structure with HPMC in low concentration was not enough to firmly block the liquid oil under high-speed centrifugation. When the HPMC concentration was increased to 1.0 wt%, the OLR decreased to 5.5%, showing good oil retention. When the PC concentration was increased from 0 wt% to 0.2 wt%, the OLR also decreased from 11.3% to 5.4%. It indicated that the increase in polysaccharide concentration had a positive effect on the oleogels. This was consistent with the findings of Espert et al. who concluded that high concentrations of polysaccharides tended to form stronger network structures, thereby enhancing the oil binding capacity [15].

### 2.5. XRD Analysis

**Figure 8:**
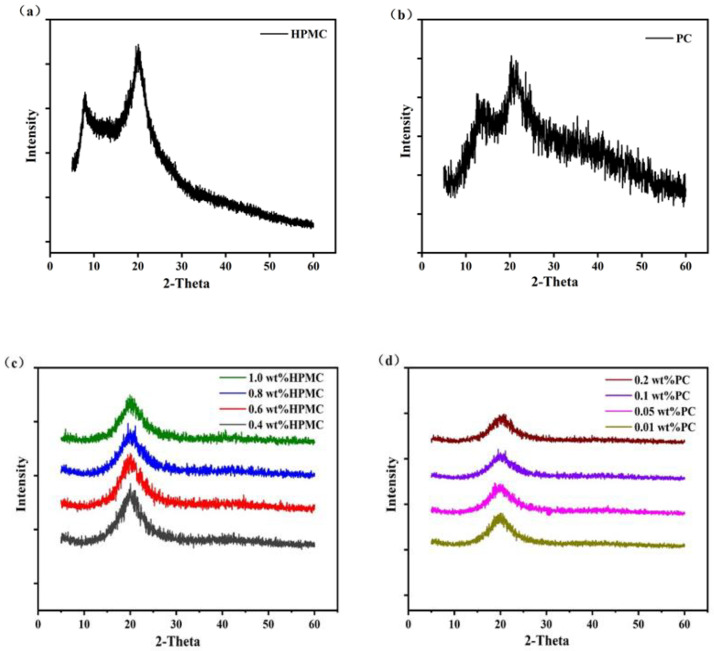
XRD patterns of HPMC powder (**a**), PC powder (**b**). XRD patterns of oleogels prepared by different HPMC concentrations and a constant PC concentration (0.1 wt%) (**c**). XRD patterns of oleogels prepared by different PC concentrations and a constant HPMC concentration (0.8 wt%) (**d**). (HPMC—Hydroxypropyl methylcellulose, PC—Pectin).

In order to understand the structure of oleogels, the internal structures of polysaccharide powder and oleogels were determined and analyzed by X-ray diffractometer (XRD). The XRD patterns of HPMC powder, PC powder, and different oleogels were shown in Figure 8. HPMC powder showed two characteristic diffraction peaks at 8.5° and 20.1° (2 θ), while PC powder showed two characteristic peaks at 14.2° and 21.8° (2 θ) [35]. In the X-ray diffraction patterns of all oleogels, the diffraction intensities of HPMC at 8.5° and PC at 14.2° (2 θ) disappeared, and no crystallization was even found. This might be because the intermolecular interaction and amorphous molecular arrangement of HPMC and PC disrupt the ordered structure present in this region, resulting in the disappearance of the peaks at 8.5° for HPMC and 14.2° (2 θ) for PC. A study found that when MC was mixed with EG, the interaction of EG with MC led to the disappearance of the peak of MC at 9° (2 θ) [36]. Moreover, a broad peak appeared around 20.5° for different oleogels, indicating that they had similar internal structures. This might be due to the extension and entanglement of the polysaccharide molecular chain, resulting in a conformational transition [37]. During the dehydration process, the interaction of HPMC and PC molecules formed a relatively ordered structure to capture the liquid oil, so that the oleogels had an ordered structure with a certain crystalline region. However, there was no obvious effect of HPMC or PC concentration on the XRD patterns of the oleogels. Meng’s study also found that HPMC and MC with different relative molecular weights also had no significant effect on the XRD patterns of oleogels [38].

## 3. Materials and Methods

### 3.1. Materials

The hydroxypropyl methylcellulose (HPMC) (28–30% methoxyl, 7.0–12% hydrox-ypropyl), with a viscosity of 400 mPa s at 2% aqueous solution at 20 °C was purchased from Shanghai Titan Scientific Co., Ltd. (Shanghai, China). Pectin from citrus peel was purchased from Sigma-Aldrich (St. Louis, MA, USA), its galacturonic acid content was more than 74.0% (in dry basis) according to the manufacturer’s instruction. The degree of methylation (DM) was 70.2 ± 0.4%. Medium-chain triglycerides (MCT) oil (with C8 and C10 carbon chain, 99% purity) were provided by Hainan Dabai Health Medicine Co., Ltd. All sample concentrations were expressed as a mass percentage (*w*/*w*, %).

### 3.2. Preparation for Samples

#### 3.2.1. Preparation of Oleogels with Different HPMC Concentrations

Firstly, both HPMC and PC solutions were prepared at 25 °C, pH 7.0, and without salt ions. Polysaccharide mixtures with different concentrations of HPMC were obtained by mixing 0.1 wt% PC with different concentrations of HPMC (0.4, 0.6, 0.8, 1.0 wt%) for 30 min. Then, 60 wt% MCT was added to the polysaccharide mixtures with different HPMC concentrations and the mixtures were sheared at 12,000 rpm/min for 2 min using a high-speed shearing homogenizer (Shanghai Huxi Industrial Co., Ltd., Shanghai, China) to obtain the emulsions. The emulsions were placed in a vacuum drying oven (Shanghai Yihengke Instrument Co., Ltd., Shanghai, China) at 70 °C for about 36 h to obtain dried products with constant weight. Then, the dried material was sheared using a pulverizer (Hefei Rongshida Co., Ltd., Hefei, China) for 6 cycles of 5 s each to obtain oleogels.

#### 3.2.2. Preparation of Oleogels with Different PC Concentrations

Polysaccharide mixtures with different concentrations of PC were obtained by mixing 0.8 wt% HPMC with different concentrations of PC (0.01, 0.05, 0.1, 0.2 wt%) for 30 min to obtain polysaccharide mixtures with different PC concentrations. The following steps were the same as above.

### 3.3. Determination of Particle Size of Emulsions

An appropriate amount of the emulsions was diluted in distilled water, and the particle size distribution and volume-average diameter (D[4,3]) of the emulsions were measured using a laser particle size distribution analyzer (Dandong Baite Instrument Co., Ltd., Tianjin, China). The refractive index of water, refractive index and light shielding ratio of the samples were set to 1.33, 1.44 and 8–12%, respectively. The reported results were the average of three parallel experiments at room temperature.

### 3.4. Microstructure of Emulsions

Optical microscopy (Xiamen Microaudie Industrial Group Co., Ltd., Xiamen, China) was used to observe the microstructure of the emulsions. A total of 10 μL of the emulsion was placed on an optical microscope slide and the oil droplets were observed and recorded by a computer-built camera with a 40× magnifying glass.

### 3.5. Rheological Measurements

The rheological properties of the emulsions and oleogels were measured using a rheometer (Thermo Fisher Scientific, MARS40, Waltham, MA, USA) with 35 mm plate geometry. The test gap between the plates was fixed at 1 mm for the emulsion and oleogel samples. During strain sweep testing, the elastic and viscous moduli were recorded at a constant frequency of 1.0 Hz with strain amplitudes ranging from 0.1–100% and 0.01–100% for emulsions and oleogels, respectively. The frequency was set from 0.1 to 10 Hz and the fixed strain was 0.5% in the frequency sweep experiment. The range of shear rate in flow measurements was set from 0.1% to 100%. Alternating shear rates of time sweep were 0.1 s^−1^ and 10 s^−1^ for the oleogel samples. All measurements above were performed 25 °C. The reported results were the average of three parallel experiments.

### 3.6. Determination of Oil Loss

Oil loss was measured according to a centrifuge method [39]. The oleogel samples were weighed (m_1_) and were centrifuged at 10,000 rpm for 15 min (4 °C) using a high-speed centrifuge (Beijing Aibeili Co., Ltd., Beijing, China). After centrifugation, free oil on the upper layer of the centrifuge tube was removed completely, and the oleogel samples after the removal of liquid oil were measured (m_2_). The oil loss was calculated by the following Equation (1).

oil loss (%) = (m_1_ − m_2_)/m_1_ × 100, (1)

### 3.7. XRD Analysis

The diffraction spectrum of the sample was measured using an X-ray diffractometer (Rigaku, Tokyo, Japan) at room temperature. The range of diffraction angles was 5°–60° (2θ), and the scanning speed of the sample was 10 deg/min. The wavelength of the X-ray radiation was 1.540593.

### 3.8. Statistical Analysis

The data were analyzed using SPSS. All experimental data were the average of three parallel experiments and the statistical significance was determined at *p* < 0.05.

## 4. Conclusions

In this work, oleogels were prepared by an emulsion template method and the effects of HPMC and PC concentrations on the formation and physicochemical properties of oleogels were investigated. The results showed that the higher concentrations of HPMC and PC exhibited better emulsifying capabilities. An increase in HPMC or PC concentrations improved the mechanical strengths of oleogels and was beneficial to reducing the oil loss of oleogels. The change of HPMC and PC concentrations had no significant effect on the X-ray diffraction pattern of oleogels. Furthermore, the release rules of free fatty acids and nutrients carried by oleogel during in vitro digestion and their controlled release capabilities of nutrients could be explored in the future.

## Data Availability

Not applicable.
